# Individual optimal attentional strategy during implicit motor learning boosts frontoparietal neural processing efficiency: A functional near‐infrared spectroscopy study

**DOI:** 10.1002/brb3.1183

**Published:** 2018-12-05

**Authors:** Takeshi Sakurada, Masahiro Hirai, Eiju Watanabe

**Affiliations:** ^1^ Functional Brain Science Laboratory, Center for Development of Advanced Medical Technology Jichi Medical University Shimotsuke Japan; ^2^ Department of Neurosurgery Jichi Medical University Shimotsuke Japan; ^3^ Present address: Department of Robotics, College of Science and Engineering Ritsumeikan University Kusatsu Japan

**Keywords:** dorsolateral prefrontal cortex, focus of attention, functional near‐infrared spectroscopy, individual differences, somatosensory association cortex, visuomotor learning

## Abstract

**Introduction:**

Optimal focus of attention is a crucial factor for improving motor learning. Most previous studies have shown that directing attention to movement outcome (external focus; EF) is more effective than directing attention to body movement itself (internal focus; IF). However, our recent studies demonstrated that the optimal attentional strategy in healthy and clinical populations varies depending on individual motor imagery ability. To explore the neurological basis underlying individual optimal attentional strategy during motor learning tasks, in the present study, we measured frontoparietal activities using functional near‐infrared spectroscopy (fNIRS).

**Methods:**

Twenty‐eight participants performed a visuomotor learning task requiring circular tracking. During the task, the participants were required to direct their attention internally or externally. The individual optimal attentional strategy was determined by comparing the after‐effect sizes between the IF and EF conditions.

**Results:**

Fifteen participants showed larger after‐effects under the EF condition (External‐dominant), whereas the others showed larger after‐effects under the IF condition (Internal‐dominant). Based on the differences in neural activities between Internal‐ and External‐dominant groups, we identified the right dorsolateral prefrontal cortex (Brodmann area 46) and right somatosensory association cortex (Brodmann area 7) as the neural bases associated with individual optimal attentional strategy during motor learning. Furthermore, we observed a significant negative correlation, that is, lower activity in these areas was associated with a larger after‐effect size under the optimal attentional strategy.

**Conclusion:**

Our findings demonstrated that more efficient neural processing in the frontoparietal area under the individual optimal attentional strategy can accelerate motor learning.

## 1 INTRODUCTION

Multiple cognitive factors can affect motor learning, such as focus of attention (Peh, Chow, & Davids, 2011; Wulf, 2013) and motor imagery (Gentili, Papaxanthis, & Pozzo, 2006; Taube, Lorch, Zeiter, & Keller, 2014). Indeed, focus of attention may be one of the most influential factors facilitating motor learning (Wulf, Shea, & Lewthwaite, 2010). Previous studies exploring the effect of focus of attention on motor learning compared two different attentional strategies, internal focus (IF) and external focus (EF). In the IF strategy, the performers directed their attention toward body movement itself, whereas the performers directed their attention toward the movement outcome in the EF strategy (Wulf, Höß, & Prinz, 1998). Most studies on motor learning have shown that the EF strategy is superior to the IF strategy in both healthy and clinical populations. The advantage of the EF strategy is explained by the constrained action hypothesis (Wulf, McNevin, & Shea, 2001). According to this hypothesis, conscious motor control in the IF strategy constrains the performer's motor system by disrupting automatic control processes. In contrast, directing attention farther away from the body can weaken this disruption. This hypothesis is supported by several empirical findings on attentional capacity demands (Wulf et al., 2001), high‐frequency movement adjustments (McNevin, Shea, & Wulf, 2003), and electromyography (EMG) during motor learning tasks (Zachry, Wulf, Mercer, & Bezodis, 2005). For example, during a free‐throw task, the EF strategy resulted not only in greater shooting accuracy but also lower EMG activity in the biceps and triceps muscles compared to the IF strategy (Zachry et al., 2005). These findings suggest that the EF strategy reduces motor‐dependent noise associated with muscle activities, thereby facilitating fine automatic motor control.

The EF strategy is considered more effective for all performers. However, a few previous studies reported that the EF strategy was not necessarily advantageous for better motor performance in healthy populations (Castaneda & Gray, 2007; Emanuel, Jarus, & Bart, 2008; Perkins‐Ceccato, Passmore, & Lee, 2003). For instance, low‐skill golfers showed better performance under IF instructions than under EF instructions (Perkins‐Ceccato et al., 2003). The lack of an EF strategy advantage was also reported in a leg stepping task for patients with stroke (Kal et al., 2015). Furthermore, our recent studies demonstrated that the EF strategy did not always lead to better motor performance in healthy and stroke populations (Sakurada, Hirai, & Watanabe, 2016; Sakurada, Nakajima, Morita, Hirai, & Watanabe, 2017). We hypothesized that individual optimal attentional strategy varies depending on the ability for motor imagery, and so we assessed the modality dominance of motor imagery using questionnaires. Like the distinct attentional strategies in motor learning, motor imagery can be divided into two modalities, kinesthetic and visual motor imageries (Guillot et al., 2009). Kinesthetic motor imagery (KI) involves simulating the feeling of muscle or joint sensations, while visual motor imagery (VI) involves visualizing one's own body movement. In our studies, the VI‐dominant group showed higher motor performance when required to direct attention externally, while the KI‐dominant group showed higher motor performance when they directed attention internally. These findings suggest that the optimal attentional strategy depends on individual motor imagery ability and that the best combination improves motor performance. Taken together, the individual's dominant sensory modality in cognitive processes can enhance the motor learning effect.

Although there have been numerous behavioral studies on focus of attention in the past two decades, only a few have examined the neural activity patterns reflecting distinct attentional strategies during motor tasks. In one functional magnetic resonance imaging (fMRI) study, primary somatosensory and motor cortices exhibited greater activation under the EF condition than the IF condition during learning of a finger movement sequence (Zentgraf et al., 2009). The authors concluded that the EF strategy enhances tactile input to somatosensory areas that connect to motor areas. Another study demonstrated that the switch of attentional focus during a finger movement task induced neural activations in the left lateral premotor cortex, left primary somatosensory cortex, and intraparietal lobule (Zimmermann et al., 2012). A more recent study investigated the effect of focus of attention on the activity of the primary motor cortex by transcranial magnetic stimulation (TMS) (Kuhn, Keller, Ruffieux, & Taube, 2016). In this study, paired‐pulse TMS was applied to evaluate short‐interval intracortical inhibition (SICI) during an isometric force control task. The inhibitory circuit had been previously shown to strongly influence motor function (Flamand, Nadeau, & Schneider, 2012; Fujiyama, Hinder, Schmidt, Garry, & Summers, 2012; Heise et al., 2013), and the results of this TMS study showed that SICI was significantly greater under the EF than the IF strategy. Thus, attentional strategy during a motor task can modulate the activity of inhibitory circuits within the primary motor cortex. Taken together, these previous neuroimaging studies suggest that attentional strategy can affect neural activity in motor‐related areas.

The motor‐related areas are connected to the frontoparietal network (Dosenbach, Fair, Cohen, Schlaggar, & Petersen, 2008) such as projecting motor error information from the cerebellum to the dorsolateral prefrontal cortex (Kelly & Strick, 2003). Moreover, the frontoparietal network also has been reported as an important region for attention control (Corbetta & Shulman, 2002; Dosenbach et al., 2008; Hu et al., 2013; Jerde & Curtis, 2013; Kehrer et al., 2015). It has been proposed that the dorsolateral prefrontal cortex maintains behavioral goals in working memory and protects them from distracting information, and the inferior parietal lobe initiates shifts of attention and maintains attention on the relevant stimulus (Ptak, 2012). Furthermore, the frontal cortex has an important role in processing internal body information such as tactile stimuli (Pleger et al., 2006) and haptic information (Kaas, Mier, & Goebel, 2007), and we recently reported that the individual capacity to process internal body information can determine the optimal attentional strategy (Sakurada et al., 2017). These findings imply that frontal cortex is a potential region where individual differences in neural activity may contribute to optimal attentional strategy during motor tasks.

In summary, our previous studies have suggested that individual optimal attentional strategy is dependent on individual motor imagery ability and that the optimal combination of these can lead to better motor performance. We interpreted this to mean that the optimal attentional strategy is associated with the sensory modality, which each individual is good at processing. However, to the best of our knowledge, no previous study has examined neural activities reflecting individual differences in optimal attentional strategy during a motor learning task. In the present study, we focused on the frontoparietal area responsible for attention control and hypothesized that neural processing capacity or efficiency in the frontoparietal area could change in relation to the individual optimal attentional strategy during a motor learning task. Thus, we investigated the relationship among motor imagery ability, optimal attentional strategy, and frontoparietal activities. For example, if individuals with a kinesthetic dominance of motor imagery are good at processing tactile or somatosensory information, we can expect that their frontoparietal area will show more efficient activity when they direct their attention to their internal body information (i.e., IF). To verify this hypothesis, we measured activities in these areas using functional near‐infrared spectroscopy (fNIRS). Participants performed a visuomotor rotation learning task in which the direction of attention was manipulated (IF or EF conditions). We expected a significant correlation between neural activities in the frontoparietal area and motor learning effect according to individual optimal attentional strategy.

## 2 METHODS

### 2.1 Participants

We recruited the students and staff of Jichi Medical University as participants. Inclusion criteria were that participants should be right‐handed, have normal or corrected‐to‐normal vision, and no medical history of diseases involving motor or cognitive dysfunction. Twenty‐eight individuals were recruited (age, 18–33 years; 14 females and 14 males). None of the participants had a notable sporting ability. Participants received an explanation about the purpose of the research and the experimental tasks involved from one of the study investigators. The laterality score as assessed by the Edinburgh Inventory (Oldfield, 1971) was 93.6 ± 9.6 (mean ± *SD*). All participants provided written informed consent prior to their participation in this study, which was conducted in accordance with the Declaration of Helsinki and approved by the Institutional Review Board of Jichi Medical University.

### 2.2 Experimental setup

#### 2.2.1 Behavioral data acquisition

**Figure 1:**
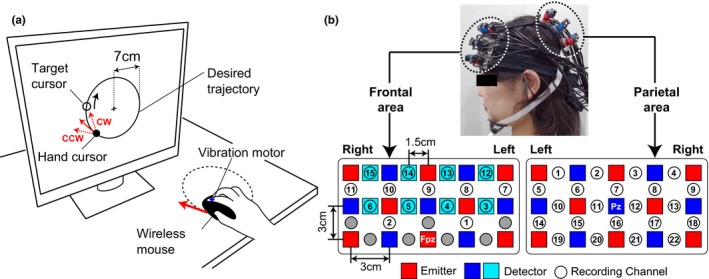
Experimental setup. (a) During task performance, the participant's hand was occluded by a small rack, preventing direct observation. (b) Probe configuration for near‐infrared spectroscopy. The probe holders were placed over the frontal and parietal areas. The spatial registration of fNIRS maps onto MNI coordinate space. Recording channels on the frontal area were numbered from lower left to upper right position, and those on the parietal area were numbered from upper left to lower right position

For the motor learning task, each participant was seated on a chair facing an LCD monitor approximately 70 cm from the participant's eyes. All visual stimuli on the monitor were programmed in Matlab (MathWorks, Natick, MA) using Cogent Toolbox software (University College London, London, UK, http://www.vislab.ucl.ac.uk/cogent.php). The participants were asked to hold a wireless computer mouse on a desk with their right hand, and we attached a vibration motor to the tip of the right index finger to present tactile stimuli. Participants could not directly see hand movements while they performed the experimental tasks as the right hand was occluded by a small rack. As shown Figure 1a, the monitor showed real‐time visual feedback of the hand movement as a hand cursor (small filled circle). The hand cursor moved synchronously with the participant's hand movement, and the position of the hand cursor on the monitor was recorded using the Cogent Toolbox with sampling at 60 Hz. The monitor also displayed a fixation cross at the center, a desired circular trajectory (large open circle; radius 7 cm) and a target cursor for tracking movements (small open circle). The participants were instructed to fixate on the cross. The ratio between actual hand movements and hand cursor movements was defined as the visual cursor gain. In this experiment, the hand cursor moved 1.2 cm on the monitor for a 1.0 cm hand movement, for a visual cursor gain of 1.2.

#### 2.2.2 fNIRS data acquisition

We used a multichannel fNIRS system (ETG‐7100, Hitachi Medical Corporation, Kashiwa, Japan) with sampling at 10 Hz. The fNIRS probes were positioned so that they covered the frontal and parietal areas (Figure 1b). We used two sets of 3 × 5 multichannel probe holders consisting of eight laser sources emitted at 695 and 830 nm (emitter; red squares in Figure 1b), and seven detecting probes (detector; blue squares in Figure 1b) arranged alternately at an inter‐probe distance of 3 cm. The midpoint between each emitter/detector pair was defined as a recording channel location (Circles in Figure 1b), and each probe holder had 22 recording channels. The probe holders were set according to the standard international 10–20 system. The probe holder on the frontal area was placed on the scalp with its lowest‐row center emitter at the participants’ Fpz position and that on the parietal area was placed on the scalp with its middle‐row center detector at the participants’ Pz position.

Functional near‐infrared spectroscopy signals reflect hemoglobin changes that originate from both cortical tissues due to brain activation and from skin blood flow. Previous studies reported that the skin blood flow can influence fNIRS signals in the frontal area during cognitive tasks (Kirilina et al., 2012; Sato et al., 2013; Takahashi et al., 2011). To eliminate the influence of skin blood flow on fNIRS signals from the frontal area, we set eight additional short detecting probes at an inter‐probe distance of 1.5 cm (light blue squares in Figure 1b) and applied multidistance independent component analysis (ICA) (Funane et al., 2014; Hirasawa et al., 2016). As it was possible to apply the multidistance ICA only to the recording channels around short detecting probes, the number of available recording channels in the probe holder on the frontal area was reduced to 15. For the spatial registration of fNIRS maps onto Montreal Neurological Institute (MNI) coordinate space, we measured scalp landmarks and all fNIRS recording channel positions using a 3D magnetic space digitizer (FASTRAK, Polhemus, USA). We then used an estimation tool without MRI (Singh, Okamoto, Dan, Jurcak, & Dan, 2005). Details on the spatial profile of recording channels are shown in Supporting Information Tables S1 and S2.

### 2.3 Procedure

#### 2.3.1 Measuring ability of motor imagery

To subjectively assess individual motor imagery ability in a manner similar to our previous study (Sakurada et al., 2016), participants completed a revised version of the Movement Imagery Questionnaire (MIQ‐R) (Hall & Martin, 1997) before the motor learning tasks. The MIQ‐R is an eight‐item self‐reported questionnaire that measures an individual's ability to imagine four simple actions: knee lift, jump, arm movement, and waist bend. The participants were required to rate the ease of imagery for each item on a seven‐point scale ranging from one (very hard to feel/see) to seven (very easy to feel/see).

#### 2.3.2 Tracking task with visuomotor rotation

We introduced three experimental conditions: no attentional instruction (NI), IF, and EF. We measured the neural activities in frontal and parietal areas by fNIRS only under the IF and EF conditions.

##### NI condition

All participants first performed a visuomotor tracking task (see next paragraph for details) under the NI condition as a practice session, and we evaluated the participants’ baseline motor performance without fNIRS recording. For the NI condition, we did not provide any instructions on how to direct attention during the visuomotor task.

**Figure 2:**
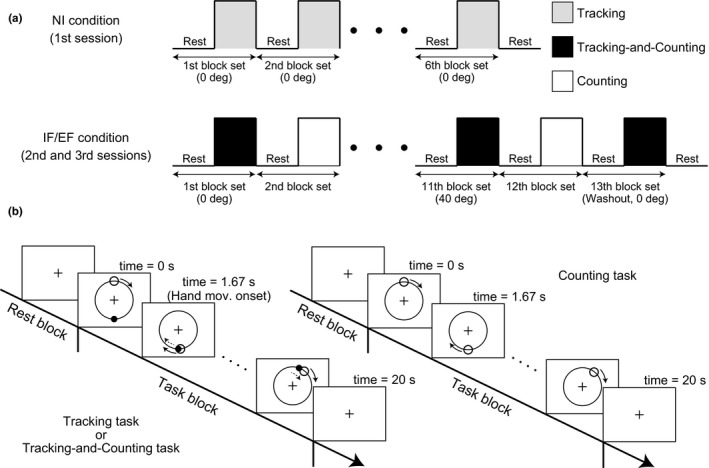
Task design. (a) Block design in the no attentional instruction (NI), internal focus (IF), and external focus (EF) conditions. Under the NI condition, the participants repeatedly performed the Tracking task in each task block. Under the IF and EF conditions, the participants alternately performed the Tracking‐and‐Counting task and the Counting task. Participants were instructed to direct their attention internally or externally on IF and EF conditions, respectively. During successive blocks, the rotation angle determining the relationship between actual hand movement and hand cursor movement increased progressively. In the final washout block set, the rotation angle was returned to 0°, and the after‐effect measured as a degree of motor learning. (b) The time sequence of one block set. In both the Tracking and the Tracking‐and‐Counting tasks, participants were required to track a target cursor with a hand cursor providing feedback

The procedure consisted of six block sets, with alternating rest (20 s) and task (20 s) blocks and an additional rest block at the end of a session (Figure 2a). The monitor displayed only the fixation cross during all rest blocks. When each task block started, the monitor showed the desired circular trajectory, the target cursor, and the hand cursor. The target cursor appeared at the top of the desired circular trajectory and began to automatically trace the circular trajectory in the clockwise direction at 0.3 Hz. The hand cursor appeared at the bottom of the desired circular trajectory, and the participants were asked to continuously move their hand to match the hand cursor with the target cursor as accurately as possible once the target cursor reached the bottom of desired circular trajectory (time = 1.67 s, Figure 2b). In the NI condition, the hand cursor always moved precisely according to the participant's hand movement (rotation angle = 0°).

In every task block, both tactile stimuli delivered by the vibration motor and visual stimuli in the form of a flickering circle around the hand cursor were presented. Although a maximum of seven stimuli was delivered in each modality with a random inter‐stimulus interval during each task block, the participants were instructed to ignore these stimuli in the NI condition.

##### IF and EF conditions

Following the NI condition, which always came first, we randomly assigned the IF and EF conditions to the second and third sessions. Under the IF condition, the participants were instructed to direct attention to their hand movements, while under the EF condition, the participants were instructed to direct attention only to the hand cursor movements on the monitor.

The second and third sessions under the IF or EF condition each consisted of 13 block sets containing alternating rest (20 s) and task (20 s) blocks. An additional rest block was inserted at the end of each session (Figure 2a). The task blocks in each session consisted of two alternating tasks, a Tracking‐and‐Counting task and a Counting task. The Tracking‐and‐Counting task was a dual task that combined the visuomotor tracking task for evaluating effects of attentional strategy on motor learning with a counting‐stimuli task for confirming whether the participants correctly directed their attention according to experimental instructions. In the Counting task, participants were required only to count external stimuli (tactile and visual as described above) without hand movement, and the monitor did not show the hand cursor. We applied this cognitive task to isolate the counting effect in the Tracking‐and‐Counting task.

In the Tracking‐and‐Counting task, visual feedback on the monitor and procedures were identical to those in the NI condition (Figure 2b). However, unlike the NI condition where visuomotor rotation angle was always 0°, we applied clockwise (CW) or counterclockwise (CCW) visuomotor transformations in which the movement direction of the hand cursor on the monitor was rotated relative to the actual hand movement direction on the desk. First, the Tracking‐and‐Counting task started under the “no rotation” setting (rotation angle = 0°), and the rotated angle was increased by 8° increments in every Tracking‐and‐Counting task block. In the 6th Tracking‐and‐Counting task block of the 11th block set, the rotation angle reached 40° (Figure 2a). Under these rotation settings, the participants were required to correctly modify their hand movements to match the hand cursor to the target cursor. However, because the rotation angle was gradually increased in every Tracking‐and‐Counting task block, the participants did not recognize the visuomotor rotation explicitly. In the 13th block set, participants performed a final Tracking‐and‐Counting task under a no rotation setting as a washout set.

In both the Tracking‐and‐Counting task and the Counting task, tactile and visual stimuli were presented, and the participants were required to count the number of tactile stimuli in the IF condition or the number of visual stimuli in the EF condition. In the IF condition, we randomly changed the number of tactile stimuli in every task block (2–7 times). Visual stimuli were also delivered, but the number was always fewer than that of tactile stimuli. On the other hand, in the EF condition, 2–7 visual stimuli and relatively fewer tactile stimuli were delivered in every task block. After changing to the rest block, the participants were asked to verbally express the number of targeted stimuli (tactile in the IF condition and visual in the EF condition). We expected that the counting requirement would help guide participants’ attention (internally or externally) according to the instruction of each session.

Half of the participants performed the visuomotor task under the IF condition with the CW setting and under the EF condition with the CCW setting, whereas the other half performed the task under the IF condition with the CCW setting and under the EF condition with the CW setting.

### 2.4 Analysis

#### 2.4.1 Ability of motor imagery

We assessed the individual ability and modality dominance of motor imagery according to the total self‐reported score on the MIQ‐R questionnaire during KI and VI. The maximum score is 28 for each modality. To subjectively characterize individual differences in modality‐specific imagery ability, we calculated a differential score by subtracting the total KI score from the VI score (∆MI = VI − KI). Participants with kinesthetic modality dominance were labeled the KI‐dominant group (∆MI < 0) and those with visual modality dominance were labeled the VI‐dominant group (∆MI > 0).

#### 2.4.2 Detection accuracy

To confirm whether the participants correctly directed their attention to the hand movements under the IF condition or to the hand cursor movements under the EF condition, we analyzed the accuracy for detecting tactile stimuli delivered by the vibration motor and visual stimuli delivered by the monitor. We calculated the proportion detected (i.e., the number of perceived stimuli to the total presented for each modality) as the detection accuracy.

#### 2.4.3 Motor performance

We defined movement error as an index of motor performance. We first calculated the distance between the target cursor and the hand cursor on the monitor for each frame and then averaged these values across a block (i.e., time = 0–20 s) as the movement error. For the NI condition, we calculated the mean movement error among six task blocks to quantify the individual's baseline motor performance. For the IF and EF conditions, in addition to the movement error from the 1st to 6th block sets, we calculated the movement error for the initial 3 s of the washout block set (termed after‐effect size) to evaluate the degree of visuomotor learning. Based on the difference in after‐effect size between the IF and EF conditions (∆AE = AE_EF _− AE_IF_), participants with positive values were classified as External‐dominant group and those with negative values as Internal‐dominant group. Furthermore, to confirm that the learning effect from the second session was successfully washed out, we also calculated the movement error for the last 3 s of the washout block set.

#### 2.4.4 fNIRS data

To analyze neural activities, we measured oxygenated hemoglobin (oxy‐Hb) signals because they are more sensitive to changes in cerebral blood flow and have higher signal‐to‐noise ratios than deoxygenated hemoglobin (deoxy‐Hb) signals (Hoshi, 2003; Strangman, Culver, Thompson, & Boas, 2002). To remove baseline drift, individual oxy‐Hb time courses from each channel were fitted to a first‐degree polynomial and high‐pass filtered using a cutoff frequency of 0.0125 Hz. We also applied low‐pass filtering using a cutoff frequency of 0.9 Hz to remove heartbeat pulsations. After removal of blocks with marked motion‐related artifacts, more than four blocks in each condition were obtained from all participants for analysis.

Raw fNIRS signals are relative values and so cannot be directly compared or averaged across channels or participants. For comparison and statistical analysis, we first converted the preprocessed oxy‐Hb signals into *z* scores using the mean value and the standard deviation of oxy‐Hb changes during the pretask period (the 10 s before starting each task block) because normalized data such as *z* scores can be averaged regardless of unit (Matsuda & Hiraki, 2006; Schroeter, Zysset, Kruggel, & Cramon, 2003). Specifically, we averaged the time‐course data of *z* scores across the same task blocks (Tracking, Tracking‐and‐Counting, or Counting) for each participant.

To explore individual differences in neural activities depending on the direction of attention, we calculated the differential time‐course data of *z* scores (∆*z*) as follows:

$$\Delta z=\left(z_{\text{EF}}^{\text{TC}}-z_{\text{EF}}^{\text{C}}\right)-\left(z_{\text{IF}}^{\text{TC}}-z_{\text{IF}}^{\text{C}}\right)$$

Here, the superscripts TC and C denote the type of experimental task (Tracking‐and‐Counting, TC; Counting, C), and the subscripts EF and IF denote the attentional condition. The time course under the Counting task was applied to remove the neural activity associated with the counting process itself. A positive ∆*z* indicated that a participant showed higher activity under the EF condition compared to the IF condition. Conversely, a negative ∆*z *indicated that a participant showed higher activity under the IF condition compared to the EF condition.

### 2.5 Statistical analysis

To compare the detection accuracy of the external stimuli, a two‐way repeated measures analysis of variance (ANOVA) was applied with task (Tracking‐and‐Counting or Counting tasks) and modality of stimulus (tactile or visual stimuli) as within‐subject factors. To evaluate the degree of visuomotor learning, a two‐way ANOVA was applied to the movement errors for the first 3 s (i.e., size of the after‐effect) and for the last 3 s of the washout block, with group (Internal‐ or External‐dominant) as a between‐subject factor and condition (IF or EF) as a within‐subject factor. A Pearson correlation coefficient (*r*) was calculated to assess the relationship between individual modality dominance of direction of attention (the motor learning effect, ∆AE) and modality dominance of motor imagery ability (∆MI).

Furthermore, to explore the between‐group differences in regional neural activities (∆*z*) between Internal‐ and External‐dominant groups, we performed frame‐by‐frame *t* tests (from −10 to 30 s). A successive time range with significant difference (*p* < 0.05, uncorrected) was considered a cluster, and statistical values in the detected clusters were corrected by cluster‐based permutation tests (Maris & Oostenveld, 2007). In addition, to assess the relationship between the behavioral trend (∆AE) and neural activity (∆*z*), a Pearson correlation coefficient (*r*) was calculated frame by frame for each channel. We considered statistical significance to be *p* < 0.05 for all tests.

## 3 RESULTS

### 3.1 Detection accuracy

All participants counted the number of tactile or visual stimuli delivered during experimental tasks with high accuracy. Detection accuracies (mean ±standard error) were 96.8% ± 1.3% for tactile stimuli during the Tracking‐and‐Counting task, 96.7% ± 1.1% for tactile stimuli during the Counting task, 93.6% ± 1.0% for visual stimuli during the Tracking‐and‐Counting task, and 94.8% ± 1.5% for visual stimuli during the Counting task. Neither the main effects of task and modality nor their interaction reached statistical significance [task: *F*(1, 27) = 0.30, *p* = 0.59, $\eta_{\text{p}}^{2}$ = 0.01, modality: *F*(1, 27) = 3.18, *p* = 0.09, $\eta_{\text{p}}^{2}$ = 0.11, task ×modality: *F*(1, 27) = 0.48, *p* = 0.50, $\eta_{\text{p}}^{2}$ *^^*= 0.02]. These high detection accuracies imply that the participants correctly and continuously directed their attention to the occluded hand under the IF condition or to the hand cursor on the monitor under the EF condition as instructed.

### 3.2 Motor performance

**Figure 3:**
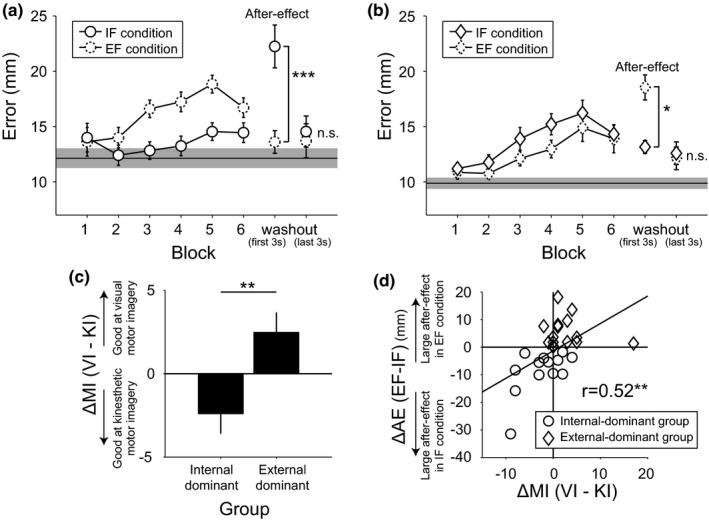
Behavioral results. (a, b) Movement error transitions under IF (internal focus, solid circles and squares) and EF (external focus, dotted circles and squares) conditions in the Internal‐dominant group (a) and the External‐dominant group (b), respectively. The thin horizontal lines and the grayed regions around the horizontal lines indicate the mean movement error and standard error under the NI condition. (c) Modality dominance of motor imagery ability in each group. (d) Significant positive correlation between individual modality dominance of motor imagery ability and differential after‐effect size. Error bars indicate the standard error. *p < 0.05, **p < 0.01, ***p < 0.001

Participants were classified into Internal‐ and External‐dominant groups based on the differential after‐effect size in the IF and EF conditions (∆AE). Individuals showing greater after‐effects (i.e., larger movement errors reflecting motor adaptation during the first 3 s of the final washout block set when rotation angle between hand and hand cursor movement was returned from 40° to 0°) in the IF condition were classified as Internal‐dominant (*n* = 13, Figure 3a) while those showing a relatively larger after‐effect under the EF condition were classified as External‐dominant (*n* = 15, Figure 3b). Regarding the transition in movement error, as the blocks progressed movement errors in the IF and EF conditions increased with visuomotor rotation angle in both Internal‐ and External‐dominant groups compared to the mean (±*SEM*) movement errors in the NI condition (Internal‐dominant group: 12.1 ± 0.9 mm, External‐dominant group: 9.9 ± 0.5 mm). However, the Internal‐dominant group showed relatively lower movement errors in the IF condition, whereas the External‐dominant group showed relatively lower movement errors in the EF condition. Regarding the after‐effect, there was a significant group ×condition interaction [*F*(1, 26) = 31.30, *p* = 0.000007, $\eta_{\text{p}}^{2}$ = 0.55], but the main effects of group and condition did not reach statistical significance [group: *F*(1, 26) = 0.93, *p* = 0.34, $\eta_{\text{p}}^{2}$ = 0.03; condition: *F*(1, 26) = 1.68, *p* = 0.21, $\eta_{\text{p}}^{2}$ = 0.06]. Post hoc analysis using the Bonferroni correction revealed that the after‐effect size under the IF condition was significantly higher than under the EF condition in the Internal‐dominant group (*p* = 0.00043). Conversely, the after‐effect size under the EF condition was significantly higher than that under the IF condition in the External‐dominant group (*p* = 0.024). The inter‐group difference under the same attentional condition reached significance only in the IF condition (IF condition: *p* = 0.0044, EF condition: *p* = 0.33). Furthermore, the movement errors for the last 3 s of the washout block set sufficiently decreased regardless of the attentional conditions or dominant groups (Bonferroni test; *p* = 0.99). Thus, we could assume that the participants could start the third session in a neutral state.

To examine the relationship between individual motor imagery ability and motor learning effect (∆AE), we compared the participants’ self‐reported MIQ‐R scores for kinesthetic and visual imageries between Internal‐dominant and External‐dominant groups. Figure 3c shows the differential modality scores on the MIQ‐R (∆MI). As expected, the External‐dominant group exhibited significantly higher VI dominance (VI score>KI score) compared to the Internal‐dominant group (*p* = 0.008, *t* test). Furthermore, we found a significant positive correlation between ∆MI and ∆AE (Figure 3d, *r* = 0.52, *p* = 0.005). This significant correlation between individual cognitive ability and motor performance is consistent with our previous reports examining healthy (Sakurada et al., 2016) and stroke populations (Sakurada et al., 2017).

### 3.3 Neural activities

**Figure 4:**
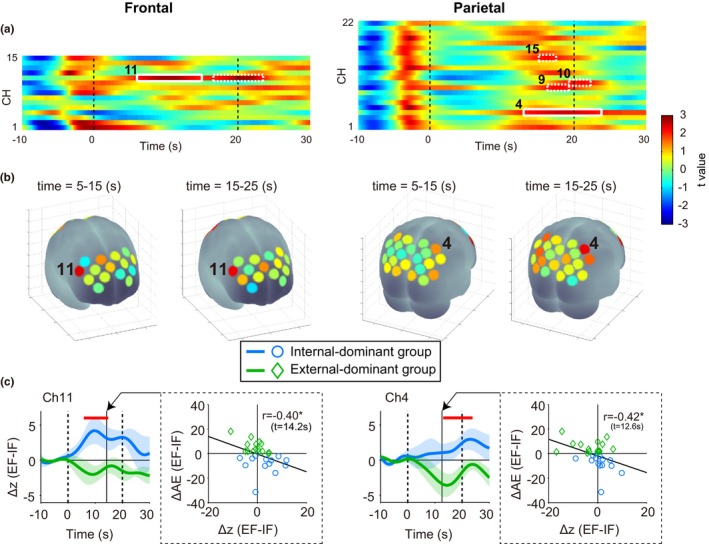
Spatiotemporal characteristics of frontal (left column) and parietal (right column) neural activities reflecting individual optimal attentional strategy. (a) Significant differences in neural activity at ch.11 of the frontal area and ch.4 of the parietal area were observed between Internal‐ and External‐dominant groups (solid white squares, *p* < 0.05, cluster‐based permutation test). (b) Spatial configuration. The *t* values of time averages are superimposed onto a brain surface. (c) The temporal profiles of differential *z* scores at channels with significant differences between dominance groups and the correlations between the differential *z* scores and differential after‐effect sizes at the time with the highest correlation coefficient. *p < 0.05

Figure 4a presents the temporal profiles of between‐group difference *t* values for fNIRS signals (∆*z*) from each channel over frontal and parietal cortex as a pseudo‐color code. Greatest differences are in red and blue. Weak significant differences were observed in ch.11 of the frontal area and ch.9, ch.10, and ch.15 of the parietal area (white dot squares, *p* < 0.05, uncorrected). Furthermore, ch.11 of the frontal area (time = 6.0–15.0 s) and ch.4 of the parietal area (time = 13.0–23.8 s) showed obvious significant differences (white solid squares, cluster‐based permutation test. ch.11 of the frontal area: *p* = 0.047, ch.4 of the parietal area: *p* = 0.016). Note that, the frontal area showed significant differences earlier than the parietal area. Figure 4b presents a statistical map of the mean *t* value within specific time windows (time = 5.0–15.0 s and time = 15.0–25.0 s) after starting the experimental task. Based on the mean *t* value, right dorsolateral prefrontal cortex (ch.11 of the frontal area) and right somatosensory association cortex (ch.4 of the parietal area) were associated with individual differences in optimal attentional strategy during motor learning. Figure 4c shows the temporal profiles of ∆*z* for significant channels. Red horizontal bars indicate clusters with successive significant differences. In these channels, the Internal‐dominant group showed significantly higher ∆*z* compared to External‐dominant group. In other words, right frontal and parietal areas showed lower activities in the Internal‐dominant group under the IF condition. Conversely, the External‐dominant group showed lower activities in these areas under the EF condition. We also found significant correlations between inter‐subject variance in neural activity (∆*z*) and those in the motor learning effect (∆AE). The strongest correlation coefficients were observed at 14.2 s in ch.11 of the frontal area (*r* = −0.40, *p* = 0.035) and at 12.6 s in ch.4 of the parietal area (*r* = −0.42, *p* = 0.025).

Additionally, we compared the temporal profiles of differential *z* scores based on the individual modality dominance of motor imagery (KI‐ vs. VI‐dominant groups). As would be expected from the significant correlation between ∆MI and ∆AE (Figure 3d), significant differences were observed between the KI‐ and VI‐dominant groups in ch.11 of the frontal area and in ch.4 of the parietal area. The KI‐dominant group showed relatively lower activity in these areas under the IF condition. Conversely, the VI‐dominant group showed lower activity under the EF condition (Supporting Information Figure S1). Note that only these two channels showed significant group differences, regardless of the type of classification (Internal‐/External‐dominant or KI‐/VI‐dominant).

## 4 DISCUSSION

Our current study found that external focus of attention did not always lead to better motor performance, in contrast with several previous studies concluding that external focus of attention is advantageous for motor learning tasks compared to internal focus of attention (Peh et al., 2011; Wulf, 2013). Rather, the current findings replicated our previous findings that individual optimal attentional strategy depends on the individual modality dominance of motor imagery (Sakurada et al., 2016, 2017). Furthermore, we found that the right dorsolateral prefrontal cortex and the right somatosensory association cortex showed lower activities under the individual optimal attentional strategy with higher motor learning effects. This finding suggests that activity in the right frontoparietal area reflects individual attention control ability during motor learning.

In accordance with our previous studies, we replicated the findings that EF did not always lead to better motor performance and that the modality dominance of motor imagery was associated with the individual optimal attentional strategy for motor learning (Sakurada et al., 2016, 2017). Several recent studies have reported findings that support these individual differences in optimal attentional strategy. For instance, in healthy populations, familiarity with attentional focus, rather than the direction of attentional focus, could be a critical factor for motor skills (Maurer & Munzert, 2013). Furthermore, children with a high conscious motor control propensity showed better performance under IF, suggesting that consistency between the direction of attentional instructions and a child's personality is a critical factor for motor skill acquisition (Tse & van Ginneken, 2017). For chronic stroke patients, EF is no longer an effective strategy (Kal et al., 2015). These findings might be related to individuals with kinesthetic motor imagery dominance, as shown in the present study, and implies that we need to classify the optimal attentional strategy for individually enhancing motor learning effects. Therefore, we should be concerned that emphasizing the EF as the most effective attentional strategy has the possibility of disadvantaging certain individuals.

Although the motor learning effect can be enhanced under the individual optimal attentional strategy, the difference between motor learning effects in the Internal‐ and External‐dominant groups under the IF condition was greater than that under the EF condition. There are two possible interpretations for this result. First is the effect of the hand cursor as the attentional target under the EF condition. Because the hand cursor was one of the visual feedbacks on the monitor, the hand cursor existed as an external attentional target that could lead to the EF strategy. Also, the hand cursor moved synchronously with the participant's hand movements, so attention toward the hand cursor could lead to IF strategy. Therefore, the difference in motor learning effects between the Internal‐ and External‐dominant groups under the EF condition might be weakened by the “mixed” focus attentional target offered by the hand cursor. Second is that an individual's cognitive ability to process internal body information, rather than external outcome information, may have a greater role in determining the optimal attentional strategy. During motor task performance, we process and integrate multimodal information from visual, proprioceptive, and vestibular inputs (Oostwoud Wijdenes & Medendorp, 2017). It can be assumed all participants naturally pay attention to visual input. On the other hand, the ability to direct attention to internal body information during a motor task can vary widely among individuals depending on a historical factor such as personal sporting experience (Sakurada et al., 2016). Hence, it is presumed that the individuals who become accustomed to processing internal body information can enhance the motor learning effect under the IF strategy without disrupting automatic motor control. Processing internal body information such as somatosensory has a crucial role in the early stage of motor skill acquisition (Bernardi, Darainy, & Ostry, 2015), so applying internal focus appropriately according to individual cognitive traits (modality dominance of motor imagery) may contribute to enhancing motor learning. Indeed, we replicated our previous findings that individual modality dominance of motor imagery correlated with the individual optimal attentional strategy (Sakurada et al., 2016, 2017). This implies that individual modality dominance is a basic cognitive characteristic and that individual dominance type pervades across multiple cognitive functions associated with motor performance such as attention control and motor imagery. Therefore, other cognitive functions with multiple modalities such as working memory may also affect task performance depending on the individual modality dominance.

Previous studies have implicated activities of motor‐related areas in focus of attention (Kuhn et al., 2016; Zentgraf et al., 2009; Zimmermann et al., 2012), while our current findings indicate that the right frontoparietal network also has an important function in determining the attentional strategy most suitable for individuals during a motor learning task. The prefrontal cortex (including the frontal eye field) and the parietal cortex are strongly interconnected by fibers passing through the superior longitudinal fasciculus (Makris et al., 2005), and the frontoparietal network does contribute to attention control (Corbetta & Shulman, 2002; Dosenbach et al., 2008; Kehrer et al., 2015). These top‐down cognitive functions enable precise motor control for high performance, so it is reasonable to suggest that frontoparietal area function is also involved in determining individual optimal attentional strategy. Supporting the possibility of the interconnection between parietal and frontal regions, we revealed temporal associations of neural activities between the two regions. We found enhanced neural activities in the frontal region reflecting individual differences at around 6.0–15.0 s followed by neural activities in the parietal region reflecting individual optimal attentional strategy at around 13.0–23.8 s. While a causal relationship remains to be verified, this delay in neural activation in the parietal region is consistent with previous reports that information flows from the frontal cortex to the parietal cortex when attention is explicitly directed (i.e., top‐down attention) (Buschman & Miller, 2010; Ptak, 2012; Scolari, Seidl‐Rathkopf, & Kastner, 2015). Furthermore, only activities in the right dorsolateral prefrontal cortex and the right somatosensory association cortex showed significant correlation with individual cognitive abilities of both motor imagery and optimal attentional strategy. These results support the notion that the frontoparietal network for processing sensory information contributes to determining individual cognitive characteristics associated with motor learning, and we propose that the modality dominance in cognitive function, such as motor imagery, is an important factor in characterizing the individual optimal attentional strategy. Individual differences in the frontoparietal network may affect the final motor outputs.

In the current study, we measured neural activities during a dual cognitive task requiring both attention control and the counting of external stimuli. Neuroimaging studies have reported that the frontal and parietal areas also have an important role in counting (Piazza, Giacomini, Bihan, & Dehaene, 2003; Piazza, Mechelli, Butterworth, & Price, 2002; Zago et al., 2010). Hence, we must consider the possibility that counting alone modulated activities in the right frontoparietal area. To address this concern, we confirmed that there was no significant difference in neural activity during the counting task between IF and EF conditions (i.e., $Z_{\text{EF}}^{\text{C}}-Z_{\text{IF}}^{\text{C}}$, data not shown). These additional analyses support the notion that activation of frontal and parietal areas reflects attention control specific to the motor task rather than for counting.

We also found that individual optimal attentional strategy associated with more efficient sensory processing in the frontoparietal area and that such efficiency is an advantage for motor learning. In particular, the right dorsolateral prefrontal cortex and the right somatosensory association cortex showed relatively lower activities under the attentional condition permitting a higher motor learning effect in a given individual. Previous studies also reported differential modulation of neural activity associated according to individual motor performance. For instance, elite professional soccer players showed lower activities in medial‐wall foot motor regions compared to football players with relatively less motor skill (Naito & Hirose, 2014). In another study, several areas associated with motor control such as primary motor cortex, supplementary motor area, premotor cortex, and superior parietal lobule showed motor skill‐dependent activity during a complex finger movement task (Krings et al., 2000). This variation in neural activity depending on motor performance is not limited to motor‐related areas. During reaching movements with visuomotor rotation, the bilateral dorsolateral prefrontal cortex showed higher activity in the initial learning phase that gradually decreased in parallel with improved motor performance as the trial proceeded (Goto et al., 2011). Therefore, the acquisition of neural processing efficiency not only in the motor‐related areas but also in the frontoparietal network is likely necessary for improved motor performance.

Although the current findings clearly indicate a relationship between individual cognitive ability, such as motor imagery and attentional strategy and the neural efficiency in the frontoparietal area, the causality between these factors is not clear. One possibility is that the individual optimal attentional strategy is a fundamental factor that can induce neural efficiency during motor learning. Another possibility is that efficient neural processing can gradually characterize the individual optimal attentional strategy during the course of motor learning. Additionally, individual cognitive ability and neural efficiency may not necessarily have a direct connection with each other. In the present study, we designed the experimental tasks to at least isolate the effect of nonfocused cognitive function (i.e., counting). However, as explained by cognitive load theory, the load of a neural process increases depending on various factors, such as task difficulty, familiarity of a task, feelings of discomfort, and so on (Sweller, 1988; Sweller, Merrienboer, & Paas, 1998). Likewise, in the case of the current task, the higher cognitive load caused by the non‐optimal attentional strategy might be indirectly linked with the relatively higher neural activities in the frontoparietal area. Further investigation is required to better understand the details of relationships, including causality in motor imagery ability, the optimal attentional strategy, and the neural activities of the frontoparietal area.

The major limitation of this study is that the motor learning task was simple compared to practical tasks in clinical rehabilitation or sports training. Thus, we need to investigate whether the frontoparietal area has the same role in daily activities. Furthermore, we focused only on the frontal and parietal areas. Neuroimaging using electroencephalography or functional magnetic resonance imaging is warranted to examine the contributions of extended brain networks to individual optimal attentional strategy.

## 5 CONCLUSIONS

The current findings suggest that individual differences in optimal attentional strategy depend on the modality dominance of motor imagery (visual vs. kinesthetic). Furthermore, the individual optimal attentional strategy is associated with greater neural processing efficiency in the frontoparietal areas. Although causality between the efficiency of the neural processes and the motor learning effects under the individual optimal attentional strategy remains unclear, we expect that a hybrid assessment protocol that combines a subjective method (motor imagery questionnaire) with an objective method (recording neural activities) could contribute to visualizing the individual neural characteristics for maximizing training effects in sports and rehabilitation medicine.

## CONFLICT OF INTEREST

The authors declare no conflicts of interest.

## References

[brb31183-bib-0001] Bernardi, N. F. , Darainy, M. , & Ostry, D. J. (2015). Somatosensory contribution to the initial stages of human motor learning. Journal of Neuroscience, 35, 14316–14326. 10.1523/JNEUROSCI.1344-15.2015 26490869 PMC4683690

[brb31183-bib-0002] Buschman, T. J. , & Miller, E. K. (2010). Shifting the spotlight of attention: Evidence for discrete computations in cognition. Frontiers in Human Neuroscience, 4, 194 10.3389/fnhum.2010.00194 21119775 PMC2990535

[brb31183-bib-0003] Castaneda, B. , & Gray, R. (2007). Effects of focus of attention on baseball batting performance in players of differing skill levels. Journal of Sport & Exercise Psychology, 29, 60–77. 10.1123/jsep.29.1.60 17556776

[brb31183-bib-0004] Corbetta, M. , & Shulman, G. L. (2002). Control of goal‐directed and stimulus‐driven attention in the brain. Nature Reviews Neuroscience, 3, 201–215. 10.1038/nrn755 11994752

[brb31183-bib-0005] Dosenbach, N. U. F. , Fair, D. A. , Cohen, A. L. , Schlaggar, B. L. , & Petersen, S. E. (2008). A dual‐networks architecture of top‐down control. Trends in Cognitive Sciences, 12, 99–105. 10.1016/j.tics.2008.01.001 18262825 PMC3632449

[brb31183-bib-0006] Emanuel, M. , Jarus, T. , & Bart, O. (2008). Effect of focus of attention and age on motor acquisition, retention, and transfer: A randomized trial. Physical Therapy, 88, 251–260. 10.2522/ptj.20060174 18042657

[brb31183-bib-0007] Flamand, V. H. , Nadeau, L. , & Schneider, C. (2012). Brain motor excitability and visuomotor coordination in 8‐year‐old children born very preterm. Clinical Neurophysiology, 123, 1191–1199. 10.1016/j.clinph.2011.09.017 22018705

[brb31183-bib-0008] Fujiyama, H. , Hinder, M. R. , Schmidt, M. W. , Garry, M. I. , & Summers, J. J. (2012). Age‐related differences in corticospinal excitability and inhibition during coordination of upper and lower limbs. Neurobiology of Aging, 33, 1484.e1–1484.e14. 10.1016/j.neurobiolaging.2011.12.019 22257984

[brb31183-bib-0009] Funane, T. , Atsumori, H. , Katura, T. , Obata, A. N. , Sato, H. , Tanikawa, Y. , … Kiguchi, M. (2014). Quantitative evaluation of deep and shallow tissue layers’ contribution to fNIRS signal using multi‐distance optodes and independent component analysis. NeuroImage, 85, 150–165. 10.1016/j.neuroimage.2013.02.026 23439443

[brb31183-bib-0010] Gentili, R. , Papaxanthis, C. , & Pozzo, T. (2006). Improvement and generalization of arm motor performance through motor imagery practice. Neuroscience, 137, 761–772. 10.1016/j.neuroscience.2005.10.013 16338093

[brb31183-bib-0011] Goto, K. , Hoshi, Y. , Sata, M. , Kawahara, M. , Takahashi, M. , & Murohashi, H. (2011). Role of the prefrontal cortex in the cognitive control of reaching movements: Near‐infrared spectroscopy study. Journal of Biomedical Optics, 16, 127003 10.1117/1.3658757 22191933

[brb31183-bib-0012] Guillot, A. , Collet, C. , Nguyen, V. A. , Malouin, F. , Richards, C. , & Doyon, J. (2009). Brain activity during visual versus kinesthetic imagery: An fMRI study. Human Brain Mapping, 30, 2157–2172. 10.1002/hbm.20658 18819106 PMC6870928

[brb31183-bib-0013] Hall, C. R. , & Martin, K. A. (1997). Measuring movement imagery abilities: A revision of the Movement Imagery Questionnaire. Journal of Mental Imagery, 21, 143–154.

[brb31183-bib-0014] Heise, K.‐F. , Zimerman, M. , Hoppe, J. , Gerloff, C. , Wegscheider, K. , & Hummel, F. C. (2013). The aging motor system as a model for plastic changes of GABA‐mediated intracortical inhibition and their behavioral relevance. The Journal of Neuroscience, 33, 9039–9049. 10.1523/JNEUROSCI.4094-12.2013 23699515 PMC6705012

[brb31183-bib-0015] Hirasawa, A. , Kaneko, T. , Tanaka, N. , Funane, T. , Kiguchi, M. , Sørensen, H. , … Ogoh, S. (2016). Near‐infrared spectroscopy determined cerebral oxygenation with eliminated skin blood flow in young males. Journal of Clinical Monitoring and Computing, 30, 243–250. 10.1007/s10877-015-9709-4 26018458

[brb31183-bib-0016] Hoshi, Y. (2003). Functional near‐infrared optical imaging: Utility and limitations in human brain mapping. Psychophysiology, 40, 511–520. 10.1111/1469-8986.00053 14570159

[brb31183-bib-0017] Hu, P. , Fan, J. , Xu, P. , Zhou, S. , Zhang, L. , Tian, Y. , & Wang, K. (2013). Attention network impairments in patients with focal frontal or parietal lesions. Neuroscience Letters, 534, 177–181. 10.1016/j.neulet.2012.12.038 23295902

[brb31183-bib-0018] Jerde, T. A. , & Curtis, C. E. (2013). Maps of space in human frontoparietal cortex. Journal of Physiology ‐ Paris, 107, 510–516. 10.1016/j.jphysparis.2013.04.002 PMC3812260 23603831

[brb31183-bib-0019] Kaas, A. L. , Van Mier, H. , & Goebel, R. (2007). The neural correlates of human working memory for haptically explored object orientations. Cerebral Cortex, 17, 1637–1649. 10.1093/cercor/bhl074 16966490

[brb31183-bib-0020] Kal, E. C. , Van Der Kamp, J. , Houdijk, H. , Groet, E. , Van Bennekom, C. A. M. , & Scherder, E. J. A. (2015). Stay focused! The effects of internal and external focus of attention on movement automaticity in patients with stroke. PLoS ONE, 10, e0136917 10.1371/journal.pone.0136917 26317437 PMC4552655

[brb31183-bib-0021] Kehrer, S. , Kraft, A. , Koch, S. P. , Kathmann, N. , Irlbacher, K. , & Brandt, S. A. (2015). Timing of spatial priming within the fronto‐parietal attention network: A TMS study. Neuropsychologia, 74, 30–36. 10.1016/j.neuropsychologia.2014.11.017 25448855

[brb31183-bib-0022] Kelly, R. M. , & Strick, P. L. (2003). Cerebellar loops with motor cortex and prefrontal cortex of a nonhuman primate. The Journal of Neuroscience, 23, 8432–8444. 10.1523/JNEUROSCI.23-23-08432.2003 12968006 PMC6740694

[brb31183-bib-0023] Kirilina, E. , Jelzow, A. , Heine, A. , Niessing, M. , Wabnitz, H. , Brühl, R. , … Tachtsidis, I. (2012). The physiological origin of task‐evoked systemic artefacts in functional near infrared spectroscopy. NeuroImage, 61, 70–81. 10.1016/j.neuroimage.2012.02.074 22426347 PMC3348501

[brb31183-bib-0024] Krings, T. , Töpper, R. , Foltys, H. , Erberich, S. , Sparing, R. , Willmes, K. , & Thron, A. (2000). Cortical activation patterns during complex motor tasks in piano players and control subjects. A functional magnetic resonance imaging study. Neuroscience Letters, 278, 189–193. 10.1016/S0304-3940(99)00930-1 10653025

[brb31183-bib-0025] Kuhn, Y. A. , Keller, M. , Ruffieux, J. , & Taube, W. (2016). Adopting an external focus of attention alters intracortical inhibition within the primary motor cortex. Acta Physiologica, 220, 289–299. 10.1111/apha.12807 27653020 PMC5484339

[brb31183-bib-0026] Makris, N. , Kennedy, D. N. , McInerney, S. , Sorensen, A. G. , Wang, R. , Caviness, V. S. , & Pandya, D. N. (2005). Segmentation of subcomponents within the superior longitudinal fascicle in humans: A quantitative, in vivo, DT‐MRI study. Cerebral Cortex, 15, 854–869. 10.1093/cercor/bhh186 15590909

[brb31183-bib-0027] Maris, E. , & Oostenveld, R. (2007). Nonparametric statistical testing of EEG‐ and MEG‐data. Journal of Neuroscience Methods, 164, 177–190. 10.1016/j.jneumeth.2007.03.024 17517438

[brb31183-bib-0028] Matsuda, G. , & Hiraki, K. (2006). Sustained decrease in oxygenated hemoglobin during video games in the dorsal prefrontal cortex: A NIRS study of children. NeuroImage, 29, 706–711. 10.1016/j.neuroimage.2005.08.019 16230030

[brb31183-bib-0029] Maurer, H. , & Munzert, J. (2013). Influence of attentional focus on skilled motor performance: Performance decrement under unfamiliar focus conditions. Human Movement Science, 32, 730–740. 10.1016/j.humov.2013.02.001 23830490

[brb31183-bib-0030] McNevin, N. H. , Shea, C. H. , & Wulf, G. (2003). Increasing the distance of an external focus of attention enhances learning. Psychological Research Psychologische Forschung, 67, 22–29. 12589447 10.1007/s00426-002-0093-6

[brb31183-bib-0031] Naito, E. , & Hirose, S. (2014). Efficient foot motor control by Neymar’s brain. Frontiers in Human Neuroscience, 8, 594. 25136312 10.3389/fnhum.2014.00594 PMC4118031

[brb31183-bib-0032] Oldfield, R. C. (1971). The assessment and analysis of handedness: The Edinburgh inventory. Neuropsychologia, 9, 97–113. 10.1016/0028-3932(71)90067-4 5146491

[brb31183-bib-0033] Oostwoud Wijdenes, L. , & Medendorp, W. P. (2017). State estimation for early feedback responses in reaching: Intramodal or multimodal? Frontiers in Integrative Neuroscience, 11, 38 10.3389/fnint.2017.00038 29311860 PMC5742230

[brb31183-bib-0034] Peh, S.‐ Y.‐C. , Chow, J. Y. , & Davids, K. (2011). Focus of attention and its impact on movement behaviour. Journal of Science and Medicine in Sport, 14, 70–78. 20727824 10.1016/j.jsams.2010.07.002

[brb31183-bib-0035] Perkins‐Ceccato, N. , Passmore, S. R. , & Lee, T. D. (2003). Effects of focus of attention depend on golfers’ skill. Journal of Sports Sciences, 21, 593–600. 10.1080/0264041031000101980 12875310

[brb31183-bib-0036] Piazza, M. , Giacomini, E. , Le Bihan, D. , & Dehaene, S. (2003). Single‐trial classification of parallel pre‐attentive and serial attentive processes using functional magnetic resonance imaging. Proceedings. Biological Sciences, 270, 1237–1245. 10.1098/rspb.2003.2356 12816636 PMC1691365

[brb31183-bib-0037] Piazza, M. , Mechelli, A. , Butterworth, B. , & Price, C. J. (2002). Are subitizing and counting implemented as separate or functionally overlapping processes? NeuroImage, 15, 435–446. 10.1006/nimg.2001.0980 11798277

[brb31183-bib-0038] Pleger, B. , Ruff, C. C. , Blankenburg, F. , Bestmann, S. , Wiech, K. , Stephan, K. E. , … Dolan, R. J. (2006). Neural coding of tactile decisions in the human prefrontal cortex. The Journal of Neuroscience, 26, 12596–12601. 10.1523/JNEUROSCI.4275-06.2006 17135421 PMC2636906

[brb31183-bib-0039] Ptak, R. (2012). The frontoparietal attention network of the human brain: Action, saliency, and a priority map of the environment. Neuroscientist, 18, 502–515. 10.1177/1073858411409051 21636849

[brb31183-bib-0040] Sakurada, T. , Hirai, M. , & Watanabe, E. (2016). Optimization of a motor learning attention‐directing strategy based on an individual’s motor imagery ability. Experimental Brain Research, 234, 301–311. 10.1007/s00221-015-4464-9 26466828

[brb31183-bib-0041] Sakurada, T. , Nakajima, T. , Morita, M. , Hirai, M. , & Watanabe, E. (2017). Improved motor performance in patients with acute stroke using the optimal individual attentional strategy. Scientific Reports, 7, 40592 10.1038/srep40592 28094320 PMC5240116

[brb31183-bib-0042] Sato, H. , Yahata, N. , Funane, T. , Takizawa, R. , Katura, T. , Atsumori, H. , … Fukuda, M. (2013). A NIRS‐fMRI investigation of prefrontal cortex activity during a working memory task. NeuroImage, 83, 158–173. 10.1016/j.neuroimage.2013.06.043 23792984

[brb31183-bib-0043] Schroeter, M. L. , Zysset, S. , Kruggel, F. , & Von Cramon, D. Y. (2003). Age dependency of the hemodynamic response as measured by functional near‐infrared spectroscopy. NeuroImage, 19, 555–564. 10.1016/S1053-8119(03)00155-1 12880787

[brb31183-bib-0044] Scolari, M. , Seidl‐Rathkopf, K. N. , & Kastner, S. (2015). Functions of the human frontoparietal attention network: Evidence from neuroimaging. Current Opinion in Behavioral Sciences, 1, 32–39. 10.1016/j.cobeha.2014.08.003 27398396 PMC4936532

[brb31183-bib-0045] Singh, A. K. , Okamoto, M. , Dan, H. , Jurcak, V. , & Dan, I. (2005). Spatial registration of multichannel multi‐subject fNIRS data to MNI space without MRI. NeuroImage, 27, 842–851. 10.1016/j.neuroimage.2005.05.019 15979346

[brb31183-bib-0046] Strangman, G. , Culver, J. P. , Thompson, J. H. , & Boas, D. A. (2002). A quantitative comparison of simultaneous BOLD fMRI and NIRS recordings during functional brain activation. NeuroImage, 17, 719–731. 10.1006/nimg.2002.1227 12377147

[brb31183-bib-0047] Sweller, J. (1988). Cognitive load during problem solving: Effects on learning. Cognitive Science, 12, 257–285. 10.1207/s15516709cog1202_4

[brb31183-bib-0048] Sweller, J. , van Merrienboer, J. J. G. , & Paas, F. G. W. C. (1998). Cognitive architecture and instructional design. Educational Psychology Review, 10, 251–296.

[brb31183-bib-0049] Takahashi, T. , Takikawa, Y. , Kawagoe, R. , Shibuya, S. , Iwano, T. , & Kitazawa, S. (2011). Influence of skin blood flow on near‐infrared spectroscopy signals measured on the forehead during a verbal fluency task. NeuroImage, 57, 991–1002. 10.1016/j.neuroimage.2011.05.012 21600294

[brb31183-bib-0050] Taube, W. , Lorch, M. , Zeiter, S. , & Keller, M. (2014). Non‐physical practice improves task performance in an unstable, perturbed environment: Motor imagery and observational balance training. Frontiers in Human Neuroscience, 8, 1–10. 10.3389/fnhum.2014.00972 25538598 PMC4255492

[brb31183-bib-0051] Tse, A. C. Y. , & van Ginneken, W. F. (2017). Children’s conscious control propensity moderates the role of attentional focus in motor skill acquisition. Psychology of Sport and Exercise, 31, 35–39. 10.1016/j.psychsport.2017.03.015

[brb31183-bib-0052] Wulf, G. (2013). Attentional focus and motor learning: A review of 15 years. International Review of Sport and Exercise Psychology, 6, 77–104. 10.1080/1750984X.2012.723728

[brb31183-bib-0053] Wulf, G. , Höß, M. , & Prinz, W. (1998). Instructions for motor learning: Differential effects of internal versus external focus of attention. Journal of Motor Behavior, 30, 169–179. 10.1080/00222899809601334 20037032

[brb31183-bib-0054] Wulf, G. , McNevin, N. , & Shea, C. H. (2001). The automaticity of complex motor skill learning as a function of attentional focus. The Quarterly Journal of Experimental Psychology A, 54, 1143–1154. 10.1080/713756012 11765737

[brb31183-bib-0055] Wulf, G. , Shea, C. , & Lewthwaite, R. (2010). Motor skill learning and performance: A review of influential factors. Medical Education, 44, 75–84. 10.1111/j.1365-2923.2009.03421.x 20078758

[brb31183-bib-0056] Zachry, T. , Wulf, G. , Mercer, J. , & Bezodis, N. (2005). Increased movement accuracy and reduced EMG activity as the result of adopting an external focus of attention. Brain Research Bulletin, 67, 304–309. 10.1016/j.brainresbull.2005.06.035 16182938

[brb31183-bib-0057] Zago, L. , Petit, L. , Mellet, E. , Joliot, M. , Mazoyer, B. , & Tzourio‐Mazoyer, N. (2010). Neural correlates of counting large numerosity. ZDM Mathematics Education, 42, 569–577. 10.1007/s11858-010-0254-9

[brb31183-bib-0058] Zentgraf, K. , Lorey, B. , Bischoff, M. , Zimmermann, K. , Stark, R. , & Munzert, J. (2009). Neural correlates of attentional focusing during finger movements: A fMRI study. Journal of Motor Behavior, 41, 535–541. 10.3200/35-08-091 19567364

[brb31183-bib-0059] Zimmermann, K. M. , Bischoff, M. , Lorey, B. , Stark, R. , Munzert, J. , & Zentgraf, K. (2012). Neural correlates of switching attentional focus during finger movements: An fMRI study. Frontiers in Psychology, 3, 555 10.3389/fpsyg.2012.00555 23444053 PMC3581438

