# Supplementary material for: Individual optimal attentional strategy during implicit motor learning boosts frontoparietal neural processing efficiency: A functional near‐infrared spectroscopy study
Source: Brain Behav. 2018 Dec 5;9(1):e01183. doi: 10.1002/brb3.1183 (PMC6346671; doi:10.1002/brb3.1183)
Supplement: Supplementary file 1 [file BRB3-9-e01183-s001.docx]

**Supplementary Information**

**Title**

Individual optimal attentional strategy during implicit motor learning boosts frontoparietal neural processing efficiency: A functional near-infrared spectroscopy study

**Authors**

Takeshi Sakurada, Masahiro Hirai and Eiju Watanabe

Supplementary Table SI: Spatial profile of each recording channel in the frontal area

| CH | MNI coordinates | | | Localization | Brodmann area | Probability |
| --- | --- | --- | --- | --- | --- | --- |
|  | *x* | *y* | *z* |  |  |  |
| 1 | -24 | 69 | 14 | Left frontopolar cortex | 10 | 1 |
| 2 | 29 | 68 | 12 | Right frontopolar cortex | 10 | 1 |
| 3 | -35 | 56 | 25 | Left frontopolar cortex | 10 | 0.89 |
|  |  |  |  | Left dorsolateral prefrontal cortex | 9 | 0.07 |
|  |  |  |  | Left dorsolateral prefrontal cortex | 46 | 0.04 |
| 4 | -10 | 66 | 28 | Left frontopolar cortex | 10 | 0.88 |
|  |  |  |  | Left dorsolateral prefrontal cortex | 9 | 0.12 |
| 5 | 17 | 66 | 28 | Right frontopolar cortex | 10 | 0.87 |
|  |  |  |  | Right dorsolateral prefrontal cortex | 9 | 0.13 |
| 6 | 41 | 56 | 24 | Right frontopolar cortex | 10 | 0.88 |
|  |  |  |  | Right dorsolateral prefrontal cortex | 46 | 0.09 |
|  |  |  |  | Right dorsolateral prefrontal cortex | 9 | 0.03 |
| 7 | -45 | 37 | 34 | Left dorsolateral prefrontal cortex | 46 | 0.53 |
|  |  |  |  | Left dorsolateral prefrontal cortex | 9 | 0.47 |
| 8 | -22 | 53 | 39 | Left dorsolateral prefrontal cortex | 9 | 0.80 |
|  |  |  |  | Left frontal eye field | 8 | 0.13 |
|  |  |  |  | Left frontopolar cortex | 10 | 0.07 |
| 9 | 4 | 58 | 40 | Right dorsolateral prefrontal cortex | 9 | 0.73 |
|  |  |  |  | Right frontopolar cortex | 10 | 0.19 |
|  |  |  |  | Right frontal eye field | 8 | 0.08 |
| 10 | 28 | 53 | 38 | Right dorsolateral prefrontal cortex | 9 | 0.79 |
|  |  |  |  | Right frontopolar cortex | 10 | 0.14 |
|  |  |  |  | Right frontal eye field | 8 | 0.07 |
| 11 | 49 | 38 | 33 | Right dorsolateral prefrontal cortex | 46 | 0.61 |
|  |  |  |  | Right dorsolateral prefrontal cortex | 9 | 0.38 |
|  |  |  |  | Right frontopolar cortex | 10 | 0.01 |
| 12 | -34 | 35 | 49 | Left frontal eye field | 8 | 0.82 |
|  |  |  |  | Left dorsolateral prefrontal cortex | 9 | 0.18 |
| 13 | -10 | 47 | 52 | Left frontal eye field | 8 | 0.93 |
|  |  |  |  | Left dorsolateral prefrontal cortex | 9 | 0.07 |
| 14 | 16 | 47 | 51 | Right frontal eye field | 8 | 0.86 |
|  |  |  |  | Right dorsolateral prefrontal cortex | 9 | 0.14 |
| 15 | 39 | 36 | 47 | Right frontal eye field | 8 | 0.69 |
|  |  |  |  | Right dorsolateral prefrontal cortex | 9 | 0.31 |

Supplementary Table SII: Spatial profile of each recording channel in the Parietal area

| CH | MNI coordinates | | | Localization | Brodmann area | Probability |
| --- | --- | --- | --- | --- | --- | --- |
|  | *x* | *y* | *z* |  |  |  |
| 1 | -33 | -60 | 67 | Left somatosensory association cortex | 7 | 0.72 |
|  |  |  |  | Left somatosensory association cortex | 5 | 0.17 |
|  |  |  |  | Left Wernicke’s area | 40 | 0.11 |
| 2 | -13 | -65 | 71 | Left somatosensory association cortex | 7 | 1 |
| 3 | 16 | -65 | 71 | Right somatosensory association cortex | 7 | 1 |
| 4 | 36 | -61 | 64 | Right somatosensory association cortex | 7 | 0.78 |
|  |  |  |  | Right Wernicke’s area | 40 | 0.12 |
|  |  |  |  | Right somatosensory association cortex | 5 | 0.10 |
| 5 | -46 | -64 | 54 | Left Wernicke’s area | 40 | 0.48 |
|  |  |  |  | Left somatosensory association cortex | 7 | 0.39 |
|  |  |  |  | Left Wernicke’s area | 39 | 0.11 |
|  |  |  |  | Left visual cortex (V3) | 19 | 0.02 |
| 6 | -24 | -74 | 61 | Left somatosensory association cortex | 7 | 1 |
| 7 | -2 | -72 | 59 | Left somatosensory association cortex | 7 | 1 |
| 8 | 25 | -74 | 60 | Right somatosensory association cortex | 7 | 1 |
| 9 | 48 | -67 | 52 | Right somatosensory association cortex | 7 | 0.42 |
|  |  |  |  | Right Wernicke’s area | 40 | 0.35 |
|  |  |  |  | Right Wernicke’s area | 39 | 0.16 |
|  |  |  |  | Right visual cortex (V3) | 19 | 0.07 |
| 10 | -36 | -79 | 49 | Left somatosensory association cortex | 7 | 0.53 |
|  |  |  |  | Left visual cortex (V3) | 19 | 0.40 |
|  |  |  |  | Left Wernicke’s area | 39 | 0.06 |
|  |  |  |  | Left Wernicke’s area | 40 | 0.01 |
| 11 | -15 | -83 | 52 | Left somatosensory association cortex | 7 | 0.72 |
|  |  |  |  | Left visual cortex (V3) | 19 | 0.28 |
| 12 | 14 | -83 | 52 | Right somatosensory association cortex | 7 | 0.72 |
|  |  |  |  | Right visual cortex (V3) | 19 | 0.28 |
| 13 | 34 | -81 | 47 | Right visual cortex (V3) | 19 | 0.52 |
|  |  |  |  | Right somatosensory association cortex | 7 | 0.47 |
|  |  |  |  | Right Wernicke’s area | 39 | 0.01 |
| 14 | -45 | -81 | 35 | Left Wernicke’s area | 39 | 0.50 |
|  |  |  |  | Left visual cortex (V3) | 19 | 0.50 |
| 15 | -26 | -89 | 40 | Left visual cortex (V3) | 19 | 0.83 |
|  |  |  |  | Left somatosensory association cortex | 7 | 0.17 |
| 16 | -4 | -89 | 39 | Left visual cortex (V3) | 19 | 0.75 |
|  |  |  |  | Left somatosensory association cortex | 7 | 0.25 |
| 17 | 23 | -91 | 39 | Right visual cortex (V3) | 19 | 0.85 |
|  |  |  |  | Right somatosensory association cortex | 7 | 0.15 |
| 18 | 44 | -83 | 30 | Right visual cortex (V3) | 19 | 0.75 |
|  |  |  |  | Right Wernicke’s area | 39 | 0.25 |
| 19 | -35 | -91 | 26 | Left visual cortex (V3) | 19 | 0.93 |
|  |  |  |  | Left Wernicke’s area | 39 | 0.05 |
|  |  |  |  | Left visual association cortex (V2) | 18 | 0.02 |
| 20 | -16 | -98 | 29 | Left visual cortex (V3) | 19 | 0.79 |
|  |  |  |  | Left visual association cortex (V2) | 18 | 0.21 |
| 21 | 11 | -98 | 29 | Right visual cortex (V3) | 19 | 0.69 |
|  |  |  |  | Right visual association cortex (V2) | 18 | 0.31 |
| 22 | 31 | -95 | 22 | Right visual cortex (V3) | 19 | 0.82 |
|  |  |  |  | Right visual association cortex (V2) | 18 | 0.18 |

Supplementary Figure S1


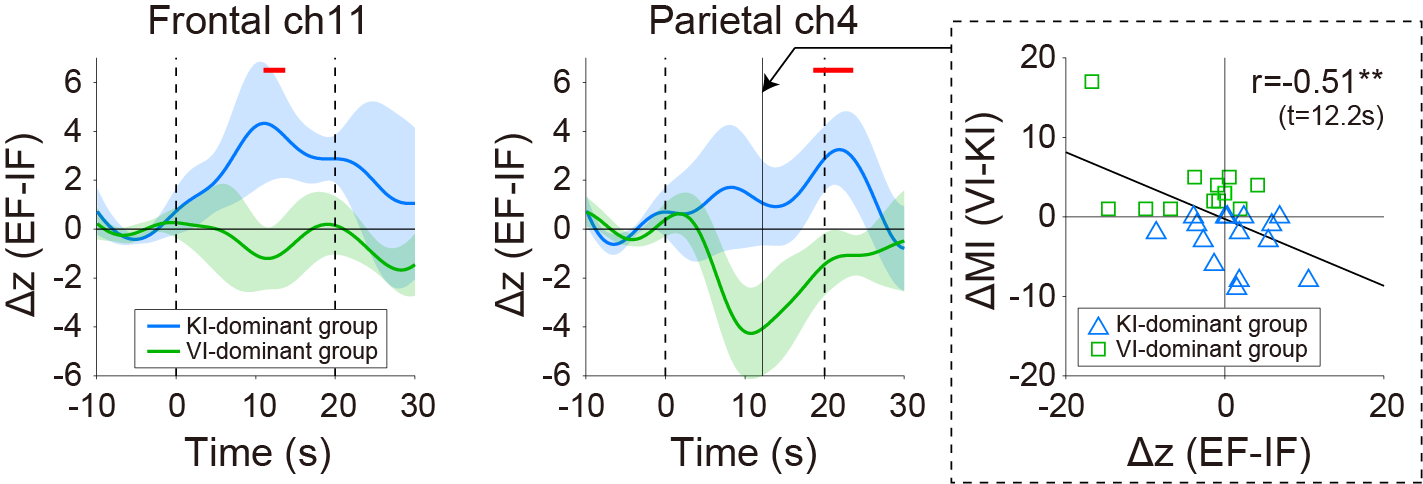


The temporal profiles of differential z scores at ch.11 of the frontal area and ch.4 of the parietal area used to classify participants into the kinesthetic motor imagery dominant group (KI-dominant group; blue lines) and the visual motor imagery dominant group (VI-dominant group; green lines). Red horizontal bars indicate clusters with successive significant differences between two groups. In both channels, we confirmed significant differences between the KI- and VI-dominant groups (*p* < 0.05; uncorrected). The KI-dominant group showed relatively lower neural activities under the IF (internal focus) condition compared to the EF (external focus) condition; however, the VI-dominant group showed the opposite trend. Furthermore, the highest significant correlation between the differential z scores and the differential motor imagery scores was observed at ch.4 of the parietal area (t = 12.2s). ***p* < 0.01
